# Spectroscopic Investigation into Oxidative Degradation of Silica-Supported Amine Sorbents for CO_2_ Capture

**DOI:** 10.1002/cssc.201100662

**Published:** 2012-06-28

**Authors:** Chakravartula S Srikanth, Steven S C Chuang

**Affiliations:** a FirstEnergy Advanced Energy Research Center, Department of Polymer Science, The University of Akron, Akron, OH 44325-3909 (USA), Fax: (+1) 330-972-5856

**Keywords:** adsorption, amines, imides, oxidation, polymers

## Abstract

Oxidative degradation characteristics of silica-supported amine sorbents with varying amounts of tetraethylenepentamine (TEPA) and polyethylene glycol (PEG; P_200_ or P_600_ represents PEG with molecular weights of 200 or 600) have been studied by IR and NMR spectroscopy. Thermal treatment of the sorbents and liquid TEPA at 100 °C for 12 h changed their color from white to yellow. The CO_2_ capture capacity of the TEPA/SiO_2_ sorbents (i.e., SiO_2_-supported TEPA with a TEPA/SiO_2_ ratio of 25:75) decreased by more than 60 %. IR and NMR spectroscopy studies showed that the yellow color of the degraded sorbents resulted from the formation of imide species. The imide species, consisting of NH associated with two C—O functional groups, were produced from the oxidation of methylene groups in TEPA. Imide species on the degraded sorbent are not capable of binding CO_2_ due to its weak basicity. The addition of P_200_ and P_600_ to the supported amine sorbents improved both their CO_2_ capture capacities and oxidative degradation resistance. IR spectroscopy results also showed that TEPA was immobilized on the SiO_2_ surface through hydrogen bonding between amine groups and the silanol groups of SiO_2_. The OH groups of PEG interact with NH_2_/NH of TEPA through hydrogen bonding. Hydrogen bonds disperse TEPA on SiO_2_ and block O_2_ from accessing TEPA for oxidation. Oxidative degradation resistance and CO_2_ capture capacity of the supported amine sorbents can be optimized through adjusting the ratio of hydroxyl to amine groups in the TEPA/PEG mixture.

## Introduction

CO_2_ emission from coal-fired power plants constituted more than 40 % of anthropogenic CO_2_ released into the atmosphere in 2010.^1^ CO_2_ separation and sequestration from coal-fired power plant flue gas is an attractive option to control CO_2_ emissions. The use of commercially available aqueous amine technology for capturing CO_2_ from flue gas could result in more than an 85 % increase in the cost of electricity.^2^ One approach to improve the aqueous amine process is to immobilize amine on porous solids, allowing surface amine sites for direct contact with CO_2_ in the flue gas. The solid amine sorbents could potentially decrease the cost of CO_2_ capture by 1) reducing the energy needed for sorbent regeneration, 2) avoiding equipment corrosion, and 3) increasing the rate of CO_2_ adsorption/absorption and desorption.

Solid amine sorbents should possess a minimum CO_2_ capture capacity of 2.0 mmol per gram of sorbent to provide a performance comparable to that of a large-scale liquid amine process.2a, 3 Solid sorbents studied so far include supported amine sorbents,2a, 3a, 4 carbon-based amine sorbents,^5^ amines coated on glass fibers,3b, 6 zeolites,^7^ SiO_2_,^7^ SBA-15,3a, 8 and MCM-41.^9^ The approaches to immobilize amines include 1) amine grafting,^[3a, 4c][3b, 6]^ using aminosilanes, and 2) physical adsorption of amines on the support surface.8a, 9a, 10 Recently, covalently tethered hyper-branched aminosilane (HAS) materials have been prepared on SBA-15, which has CO_2_ adsorption sites that can be easily regenerated.^11^ The sorbents prepared from the above approaches, despite having high capture capacities, may have limited potential for large-scale operations due to the cost of either the amines (aminosilanes) or the supports (mesoporous supports) used.

The major concern associated with amine-based sorbents is that the amines gradually degrade during thermal regeneration.^12^ Solid amine sorbents could be more prone to oxidative degradation due to the accessibility of O_2_ to the highly dispersed amines relative to limited access of O_2_ in liquid amines.

Oxidative degradation products of liquid amines, such as ethanolamines and ethylenediamines, have been identified as aldehydes and carboxylic acids.^12^ Jones et al. recently showed that secondary amines were more prone to oxidation than primary and tertiary amines when exposed to 100 % oxygen in temperature ranges of 25–135 °C.^13^ Sayari and Belmabkhout found that degradation of amine-grafted solid sorbent in the presence of CO_2_ at 105 °C led to conversion of amine to form urea and the presence of moisture in the CO_2_ gas stream slowed down the degradation.^14^ Our recent IR study showed that the addition of polyethylene glycol (PEG) to amine/SiO_2_ slowed down the formation of a thermally degraded product—carboxylate species—during CO_2_ adsorption in the presence of air and desorption in an argon gas environment.^15^ In other studies, PEG has also been used as an additive to improve the CO_2_ capture capacity of the amine-based sorbents9a, 16 and for SO_2_ capture.^17^ The oxygen in a flue gas stream, which can be trapped in the porous structure of the supported-amine sorbent, could initiate oxidative degradation during thermal desorption of adsorbed CO_2_ on the sorbent. A better understanding of the oxidative degradation process and the effect of PEG on the process could help in the development of more effective sorbents. In the present work, we used IR and NMR spectroscopy to study the structure of tetraethylenepentamine (TEPA) and PEG immobilized on SiO_2_ and to determine the oxidative degradation products. TEPA, which is a small molecule with a well-defined structure, serves as an excellent model compound to study the interaction of amines with the OH (surface silanol) groups of silica, the OH groups of PEG, and O_2_ in an oxidative environment. The results presented herein showed that oxidative degradation of the sorbent and liquid amines resulted from oxidation of methylene groups of TEPA to form imide/amide. The presence of PEG in the sorbents not only increased the CO_2_ capture capacity, but also slowed down the degradation of SiO_2_-supported amine sorbents in the oxidative (i.e., flue gas) environment. The results of this study show that the degradation of SiO_2_-supported amine strongly depends on its environment. A degradation study should be carried out in an oxygen-containing environment to emulate the practical conditions of CO_2_ capture processes.

## Results and Discussion

### Oxidative degradation study

An oxidative degradation study on silica-supported amine sorbents and TEPA/PEG liquid samples was carried out at 100 °C for 12 h. Table 1 shows the compositions of the sorbents prepared, the number of moles of amines from TEPA (NH_2_ and NH), the number of moles of hydroxyl groups from PEG present on each sorbent, and the sorbent color changes observed after the oxidative degradation studies. The extent of the color change may reflect the degree of degradation of the sorbent. All of the fresh sorbents were initially white. After oxidative degradation, the TEPA/SiO_2_ (TS 25/75) sorbent became intense yellow, whereas the PEG/SiO_2_ (P_200_S 25/75 or P_600_S 25/75) sorbents remained white. The change in the color of the TPS (TEPA/PEG/SiO_2_) sorbents varied from white to intense yellow, as shown in Table 1. The sorbents with P_200_ showed less color change than P_600_. This observation could be attributed to the higher ratio of OH to amine on P_200_ (i.e., TP_200_S) than P_600_ (i.e., TP_600_S) sorbents. The sorbents with ethylene glycol (E) and glycerol (G) changed color to intense yellow and brown, respectively. Treating liquid TEPA, PEG, and TEPA/PEG at 100 °C for 12 h also resulted in color changes and followed the same trend as those observed on TP_200_S and TP_600_S sorbents. These results showed that the presence of PEG in TS sorbents and in liquid samples slowed down the color change.

**Table 1:** 

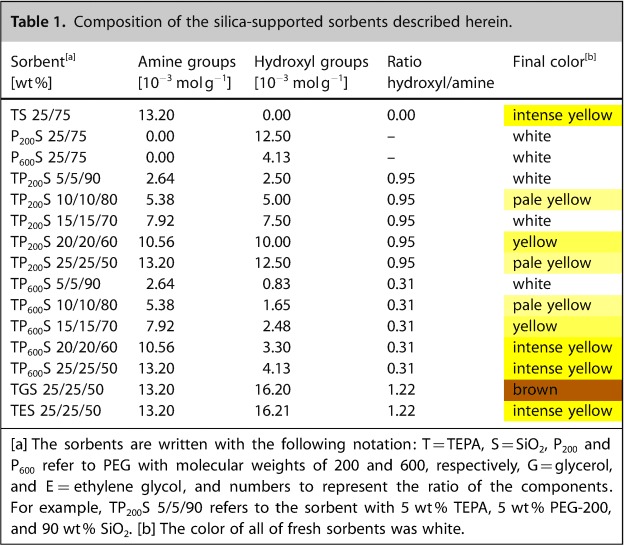

### CO_2_ capture

The sorbents were pretreated in an oven at 100 °C for 7 min and saturated in a bath with pure CO_2_ flowing at 50 cm^3^min^−1^ for 10 min over the sorbents at 25 °C. The CO_2_ capture capacity of the sorbents was measured by determining the change in weight of the sorbents before and after saturation. Table 2 contains the CO_2_ capture capacities and corresponding amine efficiencies (i.e., CO_2_/amine) of the fresh and degraded TS 25/75, TP_200_S, TP_600_S, TGS, and TES sorbents; sorbents with E and G (TES and TGS are included for comparison. A comparison of TS 25/75 with TP_200_S 25/25/50 showed that the addition of PEG to the TEPA sorbents increased both the initial capture capacity and the amine efficiency. Increasing the TEPA and PEG loading on both TP_200_S and TP_600_S sorbents increased the CO_2_ capture capacity, but decreased the amine efficiency. The decrease in the amine efficiency with TEPA and PEG loading can be attributed to the unavailability of amine sites due to agglomeration of TEPA, which causes diffusion limitations for CO_2_ to access the amine sites. The sorbents with E and G with about 30 % more hydroxyl groups than P_200_ showed higher initial capture capacities than on P_200_.

**Table 2:** CO_2_ capture capacities of fresh and degraded TS, TP_200_S, TP_600_S, TGS, and TES sorbents.

Sorbent	CO_2_ capture capacity [mmol g^−1^]
	fresh	CO_2_/amine	degraded	CO_2_/amine
TS 25/75	1.234	0.18	0.454	0.07
TP_200_S 5/5/90	0.631	0.47	0.346	0.26
TP_200_S 10/10/80	1.107	0.41	0.582	0.22
TP_200_S 15/15/70	1.585	0.39	0.868	0.21
TP_200_S 20/20/60	2.093	0.39	0.832	0.15
TP_200_S 25/25/50	2.436	0.37	1.011	0.15
TP_600_S 5/5/90	0.455	0.34	0.253	0.19
TP_600_S 10/10/80	0.842	0.32	0.448	0.16
TP_600_S 15/15/70	1.234	0.31	0.529	0.13
TP_600_S 20/20/60	1.448	0.27	0.585	0.11
TP_600_S 25/25/50	1.807	0.27	0.667	0.10
TGS^[a]^ 25/25/50	2.645	0.40	0.364	0.05
TES^[b]^ 25/25/50	2.714	0.41	0.956	0.15

[a] G=glycerol. [b] E=ethylene glycol.

CO_2_ is known to adsorb on the amine as carbamate and bicarbonate by the following reactions: in the absence of water, one mole of CO_2_ reacts with two moles of amine to form carbamate [Eq. (1)], and in the presence of water one mole of CO_2_ reacts with one mole of amine to form bicarbonate [Eq. (2)]:8b, 18],

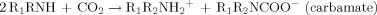
1

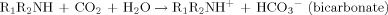
2

**Scheme 1:**
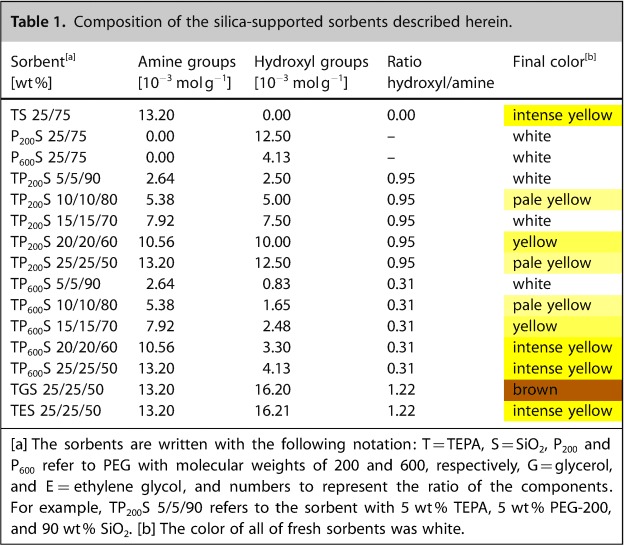
Interactions of TEPA with SiO_2_ and the formation of amide, imide, and nitrite groups by oxidative degradation.

Since CO_2_ adsorption in this study was carried out in the absence of H_2_O vapor, it is expected that CO_2_ is adsorbed in the form of carbamate on two amine sites, as shown in Equation (1). The maximum achievable amine efficiency would be 0.5. Recent studies suggested that the presence of PEG in solid amine sorbents increased the CO_2_ capture capacity by changing the rate of CO_2_ adsorption/desorption.^16^, ^18^ The CO_2_ capture results reported herein showed that the addition of PEG increased the CO_2_ capture capacities and the amine efficiencies of the sorbents; this is in good agreement with the literature.^16^, ^18^ Assuming that TEPA and PEG are adsorbed on the SiO_2_ surface by hydrogen bonding through primary amines and hydroxyl groups with the surface silanol groups, as shown in Scheme 1 below; TP_200_S 5/5/90 would occupy 30 % of the SiO_2_ surface (specific surface area 160 m^2^ g^−1^) and TP_200_S 15/15/70 would occupy 90 %. Increasing the TEPA and PEG loading beyond this value resulted in 100 % monolayer coverage and eliminated the isolated OH band at 3750 cm^−1^ (Figure 2 below). This would lead to agglomeration of TEPA and decrease the amine efficiency. However, the results in Table 2 show that amine efficiencies decrease as the loading increases from TP200S 5/5/90 to TP200S 15/15/70, indicating the occurrence of significant agglomeration and close packing of the TEPA/PEG molecules.

The extent of the decrease in CO_2_ capture capacities and amine efficiencies of the sorbents followed the order TGS>TS 25/75>TES>TP_600_S>TP_200_S after oxidative degradation. The sorbents with P_200_ also showed higher capture capacities and amine efficiencies than the TS 25/75 and TP_600_S sorbents before and after oxidative degradation. The higher capture capacities can be attributed to the presence of higher amounts of OH groups in P_200_ compared with P_600_ (Table 1). Even though the sorbents with E and G showed higher initial CO_2_ capture capacities than P_200_, they degraded more than those with P_200_ during oxidative degradation. These results indicate that the presence of P_200_ in the sorbents is more effective at slowing down the oxidative degradation of the sorbent than other additives.

Table 3 shows the CO_2_ capture capacities and amine efficiencies of amine-based sorbents reported in the literature. A maximum CO_2_ capture capacity of 4.5 mmol g^−1^ was reported on TEPA/MSF sorbent with 35 % amine efficiency.^22^ mescocellular silica foam (MSF) is a high surface area mesoporous material prepared from costly precursors. The sorbents in the present study were prepared from low-cost SiO_2_ with a surface area of 160 m^2^ g^−1^; in contrast to 600–1000 m^2^ g^−1^ for MSF, MCM-41, or SBA-15. As expected, the low surface area of the SiO_2_ support used in this study resulted in a lower CO_2_ capture than those prepared from high-surface-area supports. However, these low-surface-area supported amine sorbents have CO_2_/amine ratio (i.e., amine efficiency) values of 0.37 and 0.27 for TP_200_S 25/25/50 and TP_600_S 25/25/50, respectively (Table 2), which are comparable to the values reported in literature (Table 3). The CO_2_ capture of our low-surface-area SiO_2_ is more than 50 % of the highest reported CO_2_ capture capacity on support with more than four times the surface area of our support. These results suggest that the surface area may not be a dominating factor that governs CO_2_ capture capacity. It is noteworthy that different methods are used to measure CO_2_ capture capacity in the literature. A large number of CO_2_ capture capacity data, listed in Table 3, were determined by using the TGA method for which the weight gain in the sorbent during adsorption was measured as a function of time. Each adsorption/desorption cycle in these studies takes more than one hour, providing equilibrium CO_2_ adsorption data rather than working capacity data. These equilibrium values are expected to be significantly greater than the sorbent working capacity under practical operating conditions, where a short time period is used for adsorption/desorption. The equilibrium capture capacity of the sorbents in the present study, after keeping the sorbents in the CO_2_ bath for 30 min, was 30 % more than the values reported in Table 2. The TGA method could also overestimate the sorbent capture capacity due to the presence of H_2_O or other adsorbing contaminants in the gas stream. Non-zero steady-state CO_2_ capture capacities of TS 25/75 and TP_200_S sorbents are determined by degrading the sorbents at 100 °C and measuring the capture capacity every hour. The results (Figure S1 in the Supporting Information) showed that the degradation was fractional per cycle and TS 25/75 reached its steady state after 10 h, whereas TP_200_S sorbents reached a steady state after 24 h of oxidative degradation. These results suggest that the presence of PEG in the sorbents prevent the degradation of amines on the sorbents.

**Table 3:** Comparison of CO_2_ adsorption capacities on different amine-based sorbents reported in the literature.

Sorbent	Amine	CO_2_ capture	CO_2_/NH_2_	Cycle time	Adsorption/desorption	
	[wt %]	capacity [mmol g^−1^]		[min]	technique^[a]^	*T* [°C]	Ref.
glass fiber	30-PEI^[b]^	4.12	0.36	70	GC-TPD	80/120	3b
fly ash	60-PEI^[b, c]^	1.10	0.08	90	TGA	75/75	16
SiO_2_	54-TEPA^[c]^	2.08	0.15	30–40^[d]^	MS-TPD	30/120	15
SBA-15	50-PEI^[b]^	3.18	0.28	150	TGA	75/75	19
MCM-41	50-PEI^[b, c]^	2.50	0.22	150	TGA	75/75	9a
carbon	50-PEI^[b]^	3.06	0.27	150	TGA	75/75	19
SBA(P)	50-TEPA	3.93	0.30	150–180	fixed bed-TPD	75/100	20
Y60	50-TEPA	4.27	0.28	–	Ads.-vac.-TD	60/100	21
SBA-15	APTS	0.73	–	60^[a]^	Ads.- TPD	30/120	4a
MSF	50-TEPA	4.50	0.35	120	TGA	75/75	22
HAS	30-aziridine	1.98	0.28	>180	MS-TPD	75/130	11
SiO_2_	25-TEPA^[c]^	2.43	0.37	20	Ads.-TPD	30/100	this work

[a] GC-TPD=gas chromatography–temperature-programmed desorption, TGA=thermogravimetric analysis, MS-TPD=mass spectrometry–temperature–programmed desorption, Ads.-vac.-TD=Adsorption-vacuum desorption-thermal desorption, Ads.-TPD=Adsorption-Temperature Programmed Desorption. [b] PEI=polyethylenimine. [c] Sorbents with PEG as an additive. [c] Results from our previous work.

### IR studies on solid amine sorbents

**Figure 1:**
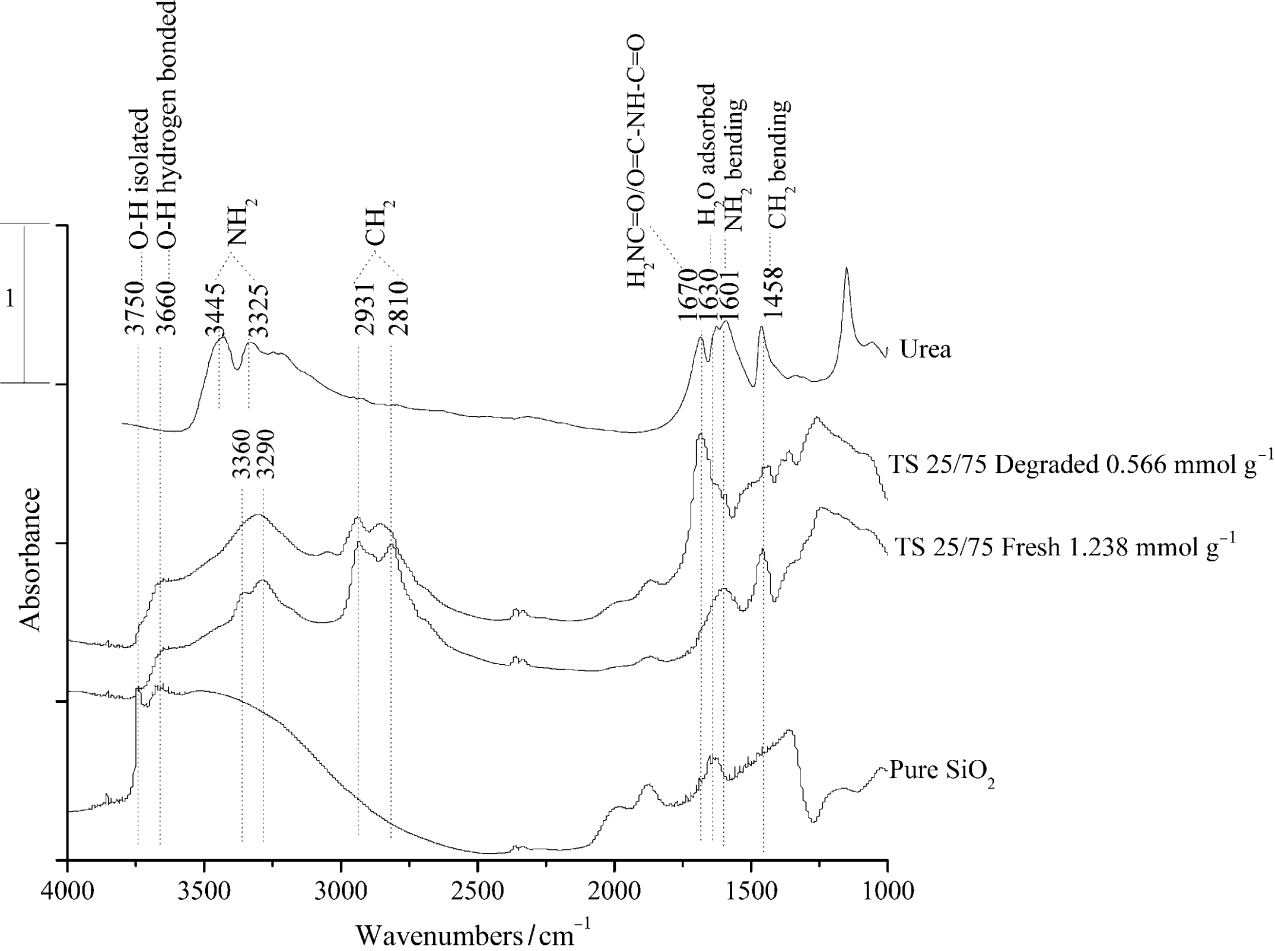
IR absorbance spectra of pure SiO_2_, fresh and degraded TS 25/75 sorbents, and urea collected by using diffuse reflectance infrared Fourier transform spectroscopy (DRIFTS) at 100 °C. Abs=log(1/*I*), in which *I* is the single beam spectra of interest.

Figure 1 shows the IR absorbance spectra of pure SiO_2_, fresh and degraded TS 25/75 (i.e., TEPA/SiO_2_) sorbents, and urea obtained by DRIFTS at 100 °C. Urea is included for comparison purposes. Pure SiO_2_ exhibited isolated O—H stretching at 3750 cm^−1^, hydrogen-bonded O—H at 3660 cm^−1^, hydrogen-bonded water at 2700–3600 cm^−1^, and a water bending vibration at 1630 cm^−1^.^23^ Impregnation of TEPA/ethanol onto SiO_2_ followed by drying produced the IR spectra of TS 25/75, which showed symmetric and asymmetric NH_2_ stretching vibrations at 3290 and 3360 cm^−1^, NH_2_ deformation of primary amines at 1601 cm^−1^, CH_2_ stretching vibrations at 2931 and 2810 cm^−1^, and bending at 1458 cm^−1^. The absence of the broad water band at 2700–3600 cm^−1^ indicates that the impregnation process displaced H_2_O from the SiO_2_ surface; the absence of the isolated O—H band at 3750 cm^−1^ suggests that these surface OH groups are bound with N in NH/NH_2_ functional groups. The IR band assignments in this study are presented in Table 4.

**Table 4:** IR band assignments.

Wavenumber [cm^−1^]	Assignment	Species	Ref.
3750	free OH groups	SiO_2_	23
3700–3000	hydrogen bonding	PEG, TEPA	3a, b, 6, 23
3660	OH-hydrogen bonded	SiO_2_	3a, b, 6, 23, 27
3500–3400	O—H stretching	PEG	17b
3360, 3290 3445, 3325	N—H stretching	TEPA	4c, 7, 8, 9b, 27, 28
2931	C—H stretching	TEPA, PEG	4c, 7, 8, 9b, 23, 28c
2810, 2880	C—H stretching	TEPA, PEG	4c, 7, 8, 9b, 23, 28c
1670	C—O stretching	amide	14, 29
1670	N—O stretching	nitrite	25
1630	H—O—H	adsorbed water	23
1601	N—H deformation of primary amine	TEPA	23, 27, 28b, c
1458	CH_2_ bending	TEPA, PEG	23
1410	C—N stretching	TEPA,	30

Degraded TS 25/75 sorbent showed 60 % less capacity than that of the fresh sorbent. The spectra of these degraded sorbents exhibited bands that were significantly different from those of urea (Figure 1). Although urea has been identified as the degradation product of aminosilane/MCM-41 in the CO_2_ stream,^14^ the absence of the IR absorbance features of urea indicated that urea was not formed in the oxidative degradation environment. The formation of urea due to reaction of CO_2_ with amines was not only associated with production of C—O, observed at 1670 cm^−1^, but should also give a peak at 1410 cm^−1^ for C—N, which was not observed in degraded sorbents of the present study. Oxidative degradation decreased the intensity of the CH_2_ bands more than the NH_2_ stretching bands. A decrease in the intensities of these bands was accompanied by the emergence of a prominent C—O band at 1670 cm^−1^ and a broad N—H band at 3290 cm^−1^. These two bands coincide with the IR spectra of an imide species consisting of NH associated with two C—O groups. Assignment of these IR bands for the yellowish degraded sorbents to imide is further supported by the yellow color of polyimide species.^24^ The yellow color could also result from oxidation of primary amines to form nitrites,^25^ which exhibit N—O stretching at 1670 cm^−1^, overlapping with the imide C—O band.^26^ The distinct asymmetric and symmetric NH_2_ bands at 3360 and 3290 cm^−1^ became a broad band centered at 3290 cm^−1^, indicating the occurrence of oxidation of these primary amine species. The spectra of the degraded sorbent also showed an increase in the intensity of the isolated OH at 3750 cm^−1^. The above IR observations suggested that TEPA on SiO_2_ was degraded through 1) oxidation of both C—H next to the secondary amines (N—H) and 2) oxidation of primary amines (NH_2_) to nitrite species. The latter appears to occur at a lesser extent than the former because the decrease in NH intensity is significantly less than C—H intensity. The NH group of the imide or amide and nitrite species is known to be a significantly weaker base than the primary amine functional group in TEPA. The weak basicity of these spices prevents them from adsorbing CO_2_. These amide/imide species are not expected to interact with isolated OH at 3750 cm^−1^.^14^ Thus, degradation of TS 25/75 sorbent would allow the isolated OH to return to its initial state, causing its IR intensity to increase.

**Figure 2:**
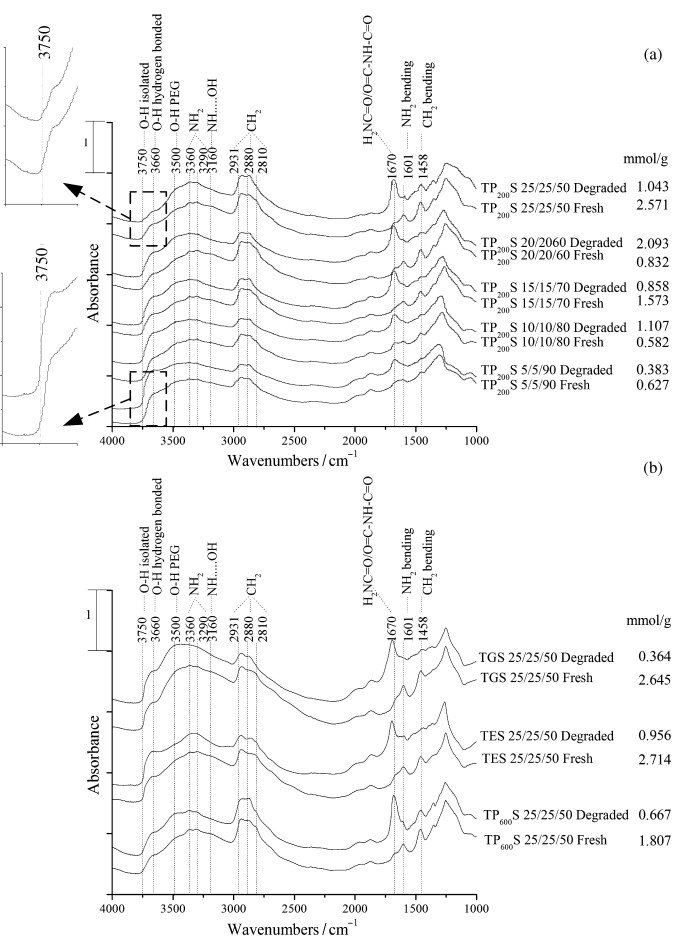
IR absorbance spectra of fresh and degraded a) TP_200_S sorbents and b) TP_600_S, TES, and TGS sorbents collected by using DRIFTS at 100 °C.

**Figure 3:**
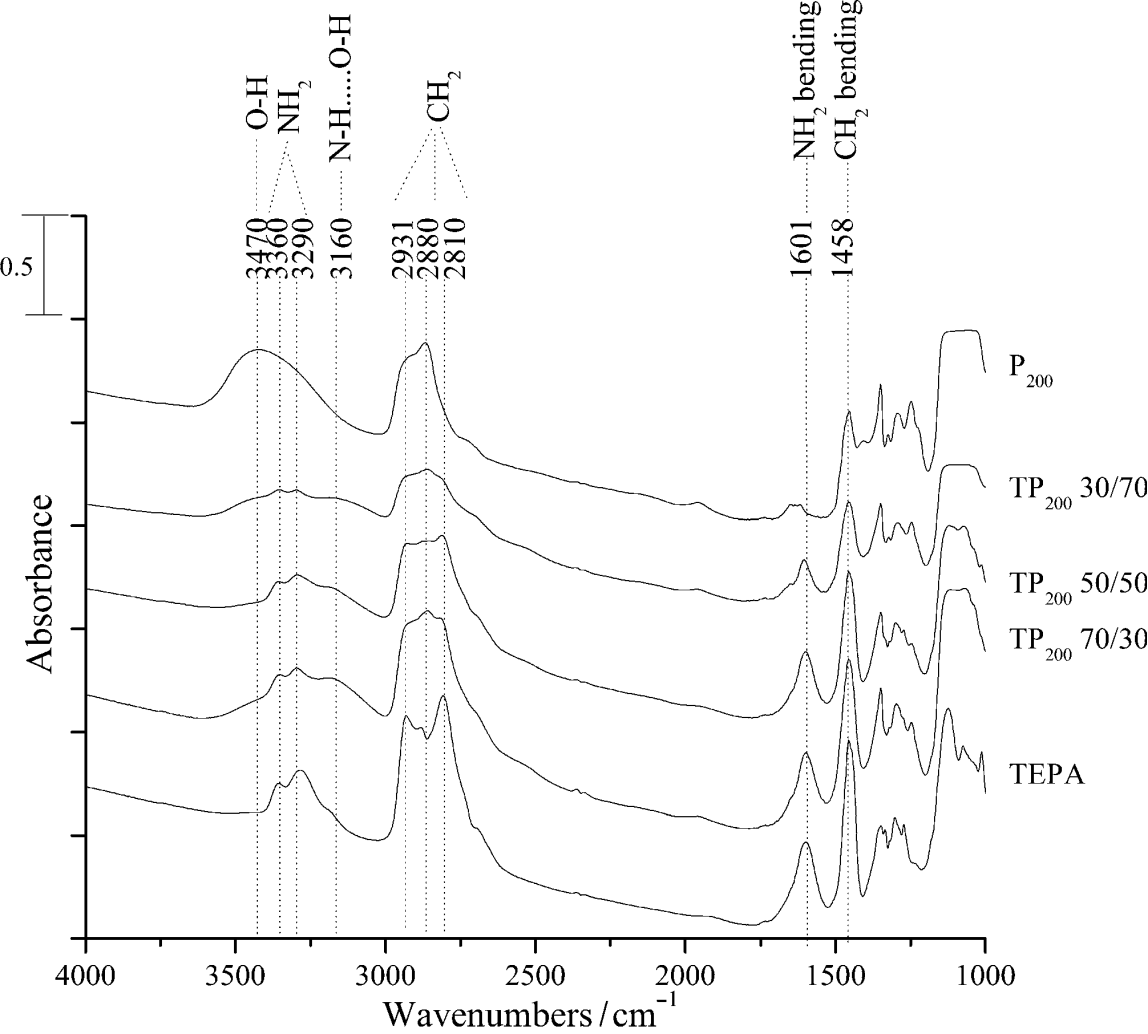
IR absorbance spectra of fresh TEPA, TP_200_, and P_200_ liquid samples with varying compositions collected by attenuated total reflectance (ATR) spectroscopy.

Figure 2 a shows the IR absorbance spectra of fresh and degraded TP_200_S sorbents, exhibiting the characteristic bands of TEPA and TP_200_ on SiO_2_. A comparison of the IR spectra of TS 25/75 and TP_200_S sorbents shows that the addition of P_200_ broadens the bands in the 3000–3600 cm^−1^ region. The amine stretching bands on TP_200_S sorbents are not as distinct as those observed on TS 25/75 sorbents. The emergence of a small hump at 3160 cm^−1^ for TP_200_S 20/20/60 suggests the formation of hydrogen bonding between the amines and OH groups of PEG. This vague band on the SiO_2_-supported sorbent became prominent in the mixture of TEPA and PEG (Figure 3 below). Figure 2 also shows that increasing the TP_200_ loading on the SiO_2_ support increased the intensities of the NH_2_ and CH_2_ bands and decreased the intensity of the shoulder at 3660 cm^−1^. This observation suggests that both TEPA and PEG displaced surface H_2_O on the SiO_2_. The decrease in the isolated OH intensity with increasing TP_200_ loading on SiO_2_ further supports the proposed interaction between isolated OH groups of silica with amines of TEPA and hydroxyl groups of PEG. Figure 2 b shows the IR absorbance spectra of fresh and degraded TP_600_S, TES, and TGS sorbents, exhibiting similar features to those observed in Figure 2 a.

The increased intensity of the 1670 cm^−1^ band for degraded sorbents is approximately proportional to the TP_200_ loading on SiO_2_ and the decrease in the intensity of N—H deformation of the primary amine at 1601 cm^−1^, CH_2_ stretchings at 2880 and 2931 cm^−1^, and the CH_2_ bending mode at 1458 cm^−1^. This observation supports our proposition that degradation occurred through oxidation of C—H bonds in TEPA to form imide species and further indicates that the extent of oxidation is proportional to the TEPA loading. The imide and nitrite species have a significantly lower basicity than the original amine and are not able to interact with CO_2_ at room temperature. These species could disrupt hydrogen bonding between those amines remaining intact and PEG OH groups on the degraded sorbents. The disruption of hydrogen bonding would allow the amines and PEG to return to hydrogen-bonding-free conditions, causing a higher intensity band of the hydroxyl groups of PEG at 3500 cm^−1^ and amine stretching bands at 3360 and 3290 cm^−1^ on the degraded sorbent relative to those on the fresh sorbents. Degradation also allows the isolated OH groups to return to their initial state, causing the IR intensity to increase, as observed in Figure 2 a (insets). However, the increase in the intensity of the band at 3750 cm^−1^ was observed more at lower loadings than those at higher loadings due to agglomeration of TEPA/PEG on the SiO_2_ surface. Sorbents with E and G (Figure 2 b) show a stronger degradation peak at 1670 cm^−1^ and loss in the hydrogen bonding band at 3160 cm^−1^ due to evaporation of the species owing to their smaller molecular size.

### IR studies on TEPA/PEG liquid samples

Figure 3 shows changes in the ATR-IR spectra of the TEPA and PEG mixture as a result of hydrogen bonding in the absence of the effect of SiO_2_. Significant broadening of the NH_2_ and OH stretching bands was observed in the 3000–3250 cm^−1^ region. Hydrogen bonding between NH_2_ groups of TEPA and OH groups of PEG produced a shoulder at around 3160 cm^−1^. The spectrum in TP_200_ 70/30 resulted from a significant fraction of PEG molecules surrounded by TEPA molecules; the spectrum in TP_200_ 30/70 resulted from TEPA molecules surrounded by PEG molecules.

**Figure 4:**
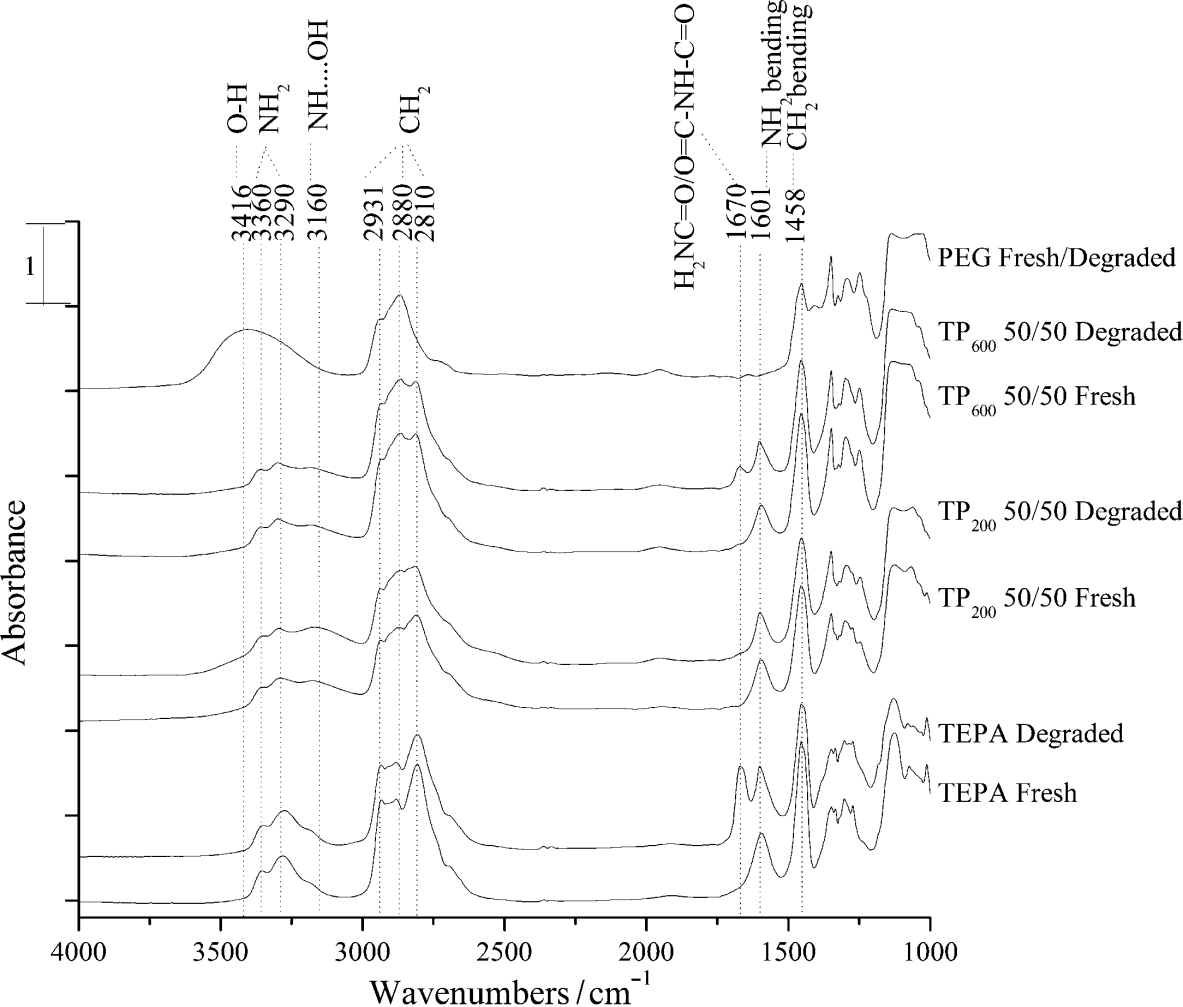
IR absorbance spectra of fresh and degraded TEPA, TP_200_ (50/50), TP_600_ (50/50), and P_200_ liquid samples collected by ATR spectroscopy. Abs=−log(*I*/*I*_o_), in which *I*_o_ is the single beam spectrum of blank ATR and *I* is the single beam spectrum of interest.

Figure 4 shows the ATR-IR absorbance spectra of fresh and degraded TEPA, TP_200_ 50/50, TP_600_ 50/50, and PEG liquid samples. Degradation of TEPA produced the C—O band at 1670 cm^−1^ with a decrease in the CH_2_ bands, suggesting that the mechanism of TEPA degradation is the same in the presence and absence of the SiO_2_ support. The significantly more intense C—O band on TEPA/SiO_2_ than that in liquid TEPA is a result of the direct exposure of TEPA to air. Oxidation in pure TEPA would require O_2_ to be dissolved in the viscous TEPA liquid. In contrast to TEPA, PEG exhibited excellent thermal stability and oxidation resistance. The fresh TP_200_ and TP_600_ solutions exhibited similar characteristic bands. Due to the low OH/amine ratio in TP_600_S sorbents, these sorbents showed less hydrogen bonding and greater degradation than TP_200_S. Less hydrogen bonding and more degradation were also observed on sorbents with G and E (i.e., TGS and TES) due to evaporation. The presence of hydrogen bonding between amine and hydroxyl groups could change the reactivity of the amines toward oxygen molecules. In addition, PEG served as an oxygen-barrier enhancer, reflecting its low permeability to O_2_.^31^. Thus, PEG enhances oxidative degradation resistance.

### ^13^C NMR spectroscopy results

**Figure 5:**
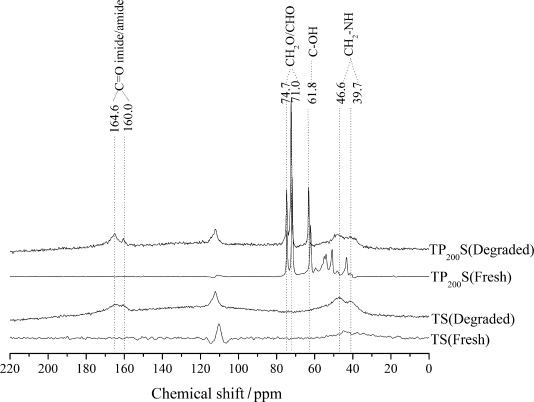
Solid-state ^13^C NMR spectra of fresh and degraded sorbents.

Figure 5 shows the ^13^C NMR spectra of fresh and degraded TS 25/75 and TP_200_S sorbents. All of the sorbents exhibited a signal at *δ*=111.9 ppm due to the background of the system and does not represent any functional group in the sample. Carbon bonded to a primary or secondary amine appeared at *δ*=46.6 and 39.7 ppm,^32^ CH_2_O in PEG resulted in the signals at *δ*=74.7 and 71.0 ppm.^33^ Carbon attached to the hydroxyl groups of PEG was observed at *δ*=61.8 ppm in the TP_200_ sorbents. The spectra of the fresh sorbents did not show any signals of carbamate,^14^ since the sorbents were pretreated at 100 °C in flowing argon (50 cc min^−1^ for 30 min) prior to NMR spectroscopy analysis to remove CO_2_ and water adsorbed from the atmosphere. Both degraded sorbents of TS and TP_200_S showed an increase in the intensity of the signals at *δ*=164.6 and 160 ppm assigned to the formation of C—O imides/amides due to the degradation of amines. These C—O signals are not likely to be associated with urea,^14^ but associated with imide/amide species. The IR spectra of the sorbents (not shown herein) after NMR spectroscopy analysis showed peaks at 1670 cm^−1^ due to prolonged scanning to get a better signal-to-noise ratio. The increase in the intensities of CH_2_NH at *δ*=44.6 and 39.7 ppm in the degraded sorbents needs further investigation. The carbon signal associated with nitrite was not observed in the NMR spectra. The ^13^C NMR spectroscopy results confirm those of the DRIFT and ATR studies, which supported the proposal that amines were degraded by oxidation of CH_2_ groups in TEPA to form imide/amide C—O groups at 100 °C in air.

### Oxidative degradation mechanism

Oxidative degradation of liquid monoethanolamine and ethylenediamines at 140 °C produced dealkylation products and small amounts of carboxylic acids.^12^ In contrast, the results described herein showed that the imide/amide species were the major degradation products formed during oxidative degradation of amines.

## Conclusions

The results of this study summarize in Scheme 1 show that amine functional groups of tetraethylenepentamine (TEPA) interact with surface hydroxyl groups of SiO_2_. Hydroxyl groups of polyethylene glycol (PEG) are also expected to interact with the surface hydroxyl groups. These interactions are evidenced by the decrease in the intensity of isolated OH groups with increasing TEPA/PEG loading. Oxidative degradation of TEPA in liquids and on SiO_2_ supports occurs through oxidation of methylene groups in TEPA to C—O, converting an amine species into imide/amide species, which leads to a color change to yellow. The weak basicity of the N—H group in imide/amide species prevents them from interacting with CO_2_ and lowers the CO_2_ capture capacities. The addition of PEG improved the CO_2_ capture capacity and oxidative resistance. The positive effect of PEG was attributed to the formation of hydrogen bonds between NH_2_ groups of TEPA and OH groups of PEG. These hydrogen-bonding interactions between amines and OH groups increased the dispersion of TEPA and slowed down the oxidation of the amine to imide species by blocking the amine sites from access to oxygen. P_200_ was more effective at slowing down the oxidative degradation of the amines than P_600_ due to more OH groups. The extent of degradation, color change, and decrease in the CO_2_ capture capacity of the sorbents are in agreement with each other.

## Experimental Section

Preparation of TPS sorbents: The specific chemicals used in the present study are tetraethylenepentamine (TEPA: T), poly(ethylene glycol) (PEG) with molecular weights 200 (P_200_) and 600 (P_600_), ethylene glycol (E), and glycerol (G), obtained from Aldrich Chemicals, USA. Silica (Brunauer–Emmett–Teller surface area 160 m^2^ g^−1^) was used as a support. Sorbents with varying weight ratios of TEPA/PEG/SiO_2_ (TPS) were prepared by impregnating 10 cm^3^ of TEPA/ethanol solution onto SiO_2_ followed by impregnating 10 cm^3^ of PEG/ethanol solution onto TEPA/SiO_2_. For example, the TP_200_S 5/5/90 sorbent was prepared by dissolving TEPA (0.25 g) or P_200_ (0.25 g) in ethanol to make a 10 cm^3^ solution for impregnation over SiO_2_ (4.5 g). The sorbents were heated at 100 °C for 15–20 min to evaporate the excess ethanol after each impregnation. TEPA/PEG liquid samples with varying compositions were prepared by mixing pure TEPA and PEG. For example, the compositions of the liquid sample TP_200_ 30/70 was obtained by mixing TEPA (0.3 g) and P_200_ (0.7 g).

CO_2_ capture and oxidative degradation: CO_2_ capture measurements for the solid sorbents were measured as follows: 1) pretreatment: sorbents were heated at 100 °C for 7 min to remove CO_2_ and H_2_O pre-adsorbed from the atmosphere; 2) CO_2_ adsorption: CO_2_ (50 cm^3^ min^−1^) was flowed for 10 min over the sorbents at 25 °C for CO_2_ adsorption; and 3) regeneration: sorbents were heated at 100 °C for 10 min for the removal of adsorbed CO_2_. The CO_2_ capture capacity of the sorbent was determined by the weight change before and after CO_2_ saturation. Short adsorption and desorption periods were used to determine the working capacity of the sorbent. The working capacity determined herein reflects the amount of captured CO_2_ in a 20 min thermal swing cycle. After regeneration the sorbents were subjected to oxidative degradation by heating the sorbent at 100 °C for 12 h in air. After oxidative degradation treatments the sorbents were described as degraded samples. The CO_2_ capture capacity of the degraded sorbents was measured in a similar way to the fresh sorbents. The liquid samples were also subjected to the same treatment to produce degraded liquid samples.

IR and NMR spectral analysis: The IR study of the fresh and degraded solid sorbents was performed ex situ by placing 50 mg of the sorbent in a DRIFT (Harrick) cell on a Nicolet-6700 FTIR spectrometer. The IR spectra of the sorbents were collected after pretreatment in flowing argon (150 cc min^−1^) at 100 °C for 10 min to remove preadsorbed water and CO_2_ from the atmosphere. The IR study of the fresh and degraded liquid samples (TEPA, P_200_, P_600_, TP_200_, and TP_600_) was performed using ATR by coating a 0.1 mm liquid layer on the ZnSe (length=4.35 cm; width=0.63 cm) window. The single beam spectra of both solid and liquid samples were collected by 32 co-added scans and a resolution of 4 cm^−1^. The absorbance spectrum was obtained by the equation Abs=log(1/*I*), in which *I* is the single beam spectrum of interest. Solid-state magic-angle spinning (MAS) ^13^C NMR spectra of the fresh and degraded sorbents were collected by using multiple-pulse direct polarization (DP) experiments at 400 MHz (Innova-400, Varian). Sorbents (≍50 mg) were packed in a Zirconia pencil rotor and spun at magic angle at 10 kHz. A relaxation time of 8 s was applied for single-pulse experiments to allow thermal equilibrium and at least 1536 scans were utilized to achieve a proper signal-to-noise ratio. Prior to NMR spectroscopy analysis the sorbents were degassed at 100 °C to remove any adsorbed CO_2_ or water from the atmosphere.

## Notes

Detailed facts of importance to specialist readers are published as ”Supporting Information”. Such documents are peer-reviewed, but not copy-edited or typeset. They are made available as submitted by the authors.

## References

[1] DOE/NETL (2010). http://www.netl.doe.gov/technologies/carbon_seq/refshelf/CCSRoadmap.pdf.

[2a] Gray ML, Hoffman JS, Hreha DC, Fauth DJ, Hedges SW, Champagne KJ, Pennline HW (2009). Energy Fuels.

[2b] Kittel J, Idem R, Gelowitz D, Tontiwachwuthikul P, Parrain G, Bonneau A (2009). Energy Procedia.

[3a] Chang ACC, Chuang SSC, Gray M, Soong Y (2003). Energy Fuels.

[3b] Li P, Ge B, Zhang S, Chen S, Zhang Q, Zhao Y (2008). Langmuir.

[3c] Zhao H, Hu J, Wang J, Zhou L, Liu H (2007). Acta Physico-Chimica Sinica.

[4a] Khatri RA, Chuang SSC, Soong Y, Gray M (2006). Energy Fuels.

[4b] Plaza MG, Pevida C, Arias B, Fermoso J, Arenillas A, Rubiera F, Pis JJ (2008). J. Therm. Anal. Calorim.

[4c] Khatri RA, Chuang SSC, Soong Y, Gray M (2005). Ind. Eng. Chem. Res.

[5a] Gray ML, Soong Y, Champagne KJ, Baltrus J, Stevens RW, Toochinda P, Chuang SSC (2004). Sep. Purif. Technol.

[5b] Su F, Lu C, Cnen W, Bai H, Hwang JF (2009). Sci. Total Environ.

[6] Li P, Zhang S, Chen S, Zhang Q, Pan J, Ge B (2008). J. Appl. Polym. Sci.

[7] Fisher II JC, Tanthana J, Chuang SSC (2009). Environ. Prog. Sustainable Energy.

[8a] Wang X, Schwartz V, Clark JC, Ma X, Overbury SH, Xu X, Song C (2009). J. Phys. Chem. C.

[8b] Zheng F, Tran DN, Busche BJ, Fryxell GE, Addleman RS, Zemanian TS, Aardahl CL (2005). Ind. Eng. Chem. Res.

[9a] Xu X, Song C, Andresen JM, Miller BG, Scaroni AW (2003). Microporous Mesoporous Mater.

[9b] Huang HY, Yang RT, Chinn D, Munson CL (2003). Ind. Eng. Chem. Res.

[10] Ma X, Wang X, Song C (2009). J. Am. Chem. Soc.

[11] Hicks JC, Drese JH, Fauth DJ, Gray MML, Qi G, Jones CW (2008). J. Am. Chem. Soc.

[12] Lepaumier H, Picq D, Carrette P-L (2009). Ind. Eng. Chem. Res.

[13a] Bollini P, Didas SA, Jones CW (2011). J. Mater. Chem.

[13b] Bollini P, Choi S, Drese JH, Jones CW (2011). Energy Fuels.

[14] Sayari A, Belmabkhout Y (2010). J. Am. Chem. Soc.

[15] Tanthana J, Chuang SSC (2010). ChemSusChem.

[16] Arenillas A, Smith KM, Drage TC, Snape CE (2005). Fuel.

[17a] Wang X, Ma X, Zhao S, Wang B, Song C (2009). Energy Environ. Sci.

[17b] Zhang J, Han F, Wei X, Shui L, Gong H, Zhang P (2010). Ind. Eng. Chem. Res.

[18] Satyapal S, Filburn T, Trela J, Strange J (2001). Energy Fuels.

[19] Wang D, Sentorun-Shalaby C, Ma X, Song C (2011). Energy Fuels.

[20] Yue MB, Chun Y, Cao Y, Dong X, Zhu JH (2006). Adv. Funct. Mater.

[21] Su F, Lu C, Kuo SC, Zeng W (2010). Energy Fuels.

[22] Liu SH, Wu CH, Lee HK, Liu SB (2010). Top. Catal.

[23] Knofel C, Martin C, Hornebecq V, Llewellyn PL (2009). J. Phys. Chem. C.

[24] Yang CP, Su YY, Wen SJ, Hsiao SH (2006). Polymer.

[25] Sexton AJ, Rochelle GT (2009). Int. J. Greenhouse Gas Control.

[26] McLaughlin RP, Donald WA, Jitjai D, Zhang Y (2007). Spectrochim. Acta, Part A.

[27] Hiyoshi N, Yogo K, Yashima T (2005). Microporous Mesoporous Mater.

[28a] Rocher N, Frech R (2007). J. Phys. Chem. A.

[28b] Pohle W, Gauger DR (2009). J. Mol. Struct.

[28c] Jackson P, Robinson K, Puxty G, Attalla M (2009). Energy Procedia.

[29] Madhurambal G, Mariappan M, Mojumdar SC (2010). J. Therm. Anal. Calorim.

[30a] Langer J, Schrader B, Bastian V, Jacob E (1995). Fresenius J. Anal. Chem.

[30b] Silverstein RM, Webstar FX, Kiemle DJ (2005). Spectrometric Identification of Organic Compounds.

[31] Tihminlioglu F, Atik ID, Ozen B (2010). J. Food Eng.

[32] Böttinger W, Maiwald M, Hasse H (2008). Fluid Phase Equilib.

[33] Heald C, Stolnik S, Kujawinski K, De Matteis C, Garnett M, Illum L, Davis S, Purkiss S, Barlow R, Gellert P (2002). Langmuir.

