# Supplementary material for: Spectroscopic Investigation into Oxidative Degradation of Silica-Supported Amine Sorbents for CO2 Capture
Source: ChemSusChem. 2012 Jun 28;5(8):1435–42. doi: 10.1002/cssc.201100662 (PMC3561697; doi:10.1002/cssc.201100662)
Supplement: Supplementary file 1 [file cssc0005-1435-sd1.pdf]

CHEMISTRY & SUSTAINABILITY
CHEMSUSCHEM
ENERGY & MATERIALS

Supporting Information

© Copyright Wiley-VCH Verlag GmbH & Co. KGaA, 69451 Weinheim, 2012

Spectroscopic Investigation into Oxidative Degradation of Silica-Supported Amine Sorbents for CO_2 Capture

Chakravartula S. Srikanth and Steven S. C. Chuang*[a]

cssc_201100662_sm_miscellaneous_information.pdf

Non-zero steady state CO_2 capture capacities

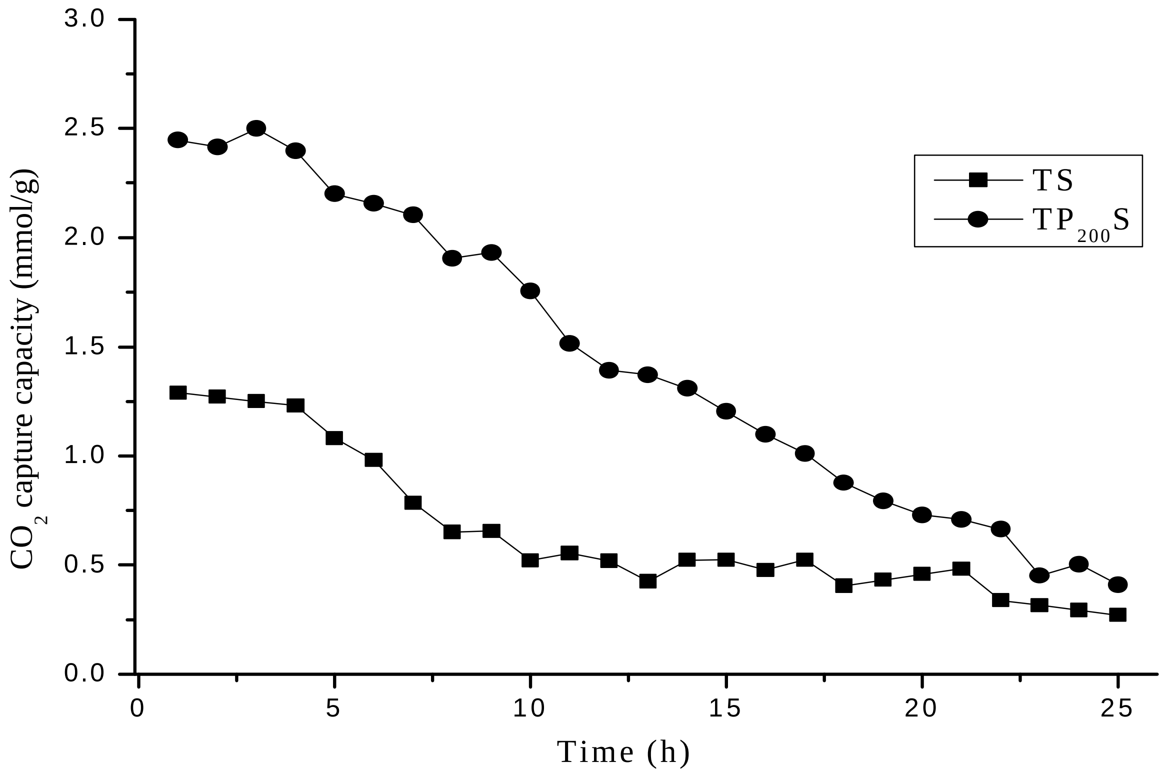

Figure S1 Non-zero steady state CO_2 capture capacity of TS 25/75 and $TP_{200}S$ sorbents

Non-zero steady state CO_2 capture capacity of TS 25/75 and $TP_{200}S$ sorbents are determined by degrading the sorbents at 100 ^{o}C and measuring the capture capacity at every hour. The change in the capture capacity of the sorbents is shown in Figure S1. The results showed that the degradation was fractional loss per cycle and TS 25/75 reached its steady state after 10 h, whereas $TP_{200}S$ sorbents reached after 24 h of oxidative degradation. These results suggest that presence of PEG in the sorbents prevent the degradation of amines on the sorbents.
